# Factors associated with long hospitalisation for psychotic disorder patients in an acute ward: Tertiary care hospital

**DOI:** 10.4102/sajpsychiatry.v30i0.2049

**Published:** 2024-04-23

**Authors:** Tshepiso I. Paliweni-Zwane, Lucas N. Modisane, Gerhard P. Grobler

**Affiliations:** 1 Department of Psychiatry, Faculty of Health Sciences, School of Medicine, University of Pretoria, Pretoria, South Africa

**Keywords:** mental health, psychiatry, acute psychiatry, length of stay, inpatient

## Abstract

**Background:**

The average length of stay is often used to indicate health system efficiency; shorter stays are associated with reduced costs. In South Africa, mental healthcare expenditure is spent on inpatient care.

**Aim:**

To identify factors associated with a long stay in an acute psychiatric unit.

**Setting:**

A tertiary hospital.

**Methods:**

A case-control study review of inpatients diagnosed with psychotic symptoms was used. Sample was divided into two groups, length of stay (LOS) (LOS greater than 21 days, LOS less than 14 days). Total of 82 patients were divided into short stay group (SSG, *n* = 23) and long stay group (LSG) (*n* = 59). A comparison of demographic, clinical and system variables was conducted.

**Results:**

In demographics, LSG had fewer men compared to SSG (78.3%) and differed statistically from LSG with *p* = 0.05. Long stay groups were older in comparison to SSG with a *p* = 0.02. Illicit substance use in LSG was 44.1% and statistically less than SSG (73.91%; *p* = 0.02). A high proportion of LSG had medical or surgical and psychiatric comorbidities (67.8%) compared to SSG (43.5%) (*p* = 0.04). A total of 95% patients in SSG had family support.

**Conclusion:**

Longer stay was found to be associated with older females with primary psychotic disorders. Comorbidities with less availability of family support were associated with younger males presenting with psychotic symptoms that may be related to illicit substances that respond to rapid stabilisation.

**Contribution:**

Active surveillance of medical comorbidities amongst older female patients is necessary for early liaison services to reduce their length of stay.

## Introduction

The number of beds available per unit of population is an important indicator of the level of inpatient mental healthcare capacity in a community.^1^ According to the World Health Organization (WHO), based on waiting and vacancy list, using social indicators that take into account specific local mental healthcare conditions, the sub-Saharan Africa and South East Asia have the fewest psychiatric beds per population, which fall well below the international standard.^1^ Unfortunately, the burden of mental illness is increasing with neuropsychiatric disorders, with the results of 2019 Global Burden of Diseases indicating that mental disorders are among the top 10 leading causes of burden worldwide, which suggests no global reduction in the burden since 1990.^2^ Recently, the national mental health policy framework and strategic planning 2013–2020 report highlighted that the psychiatry sector continues to be underfunded and under-resourced compared to other health conditions in South Africa.^2^ The introduction of deinstitutionalisation and shortening of the duration of admission could be helpful.^2^

Length of stay (LOS) in hospital encompasses the number of days between admissions and discharge.^3^ Average length of stay (ALOS) is often used to indicate health system efficiency because shorter stays are associated with reduced costs.^4^ Average length of stay is widely used in high-income countries and determinants of LOS are well described in the international literature.^5^ In general, ALOS for mental healthcare services in high-income countries is approximately half that of low- and middle-income countries (LMICs).^6^ High-income countries tend to have better community-based mental healthcare services that can provide continuity of care and reduce ALOS in acute units.^7^

In Africa, ALOS in psychiatric units in Ethiopia and Malawi has been reported as 22 days, while Nigerian psychiatric wards had an ALOS of 23 and 28 days in two acute psychiatric units.^5,7^ In psychiatry wards, the longest LOS is usually associated with psychosis, in particular schizophrenia, while patients with personality disorders and substance use disorders usually have shorter hospitalisations.^3,6,8^ Patients with substance-induced psychotic symptoms are usually treated rapidly to control the acute symptoms and discharged, while psychotic schizophrenia patients are often admitted for longer days as they may pose a threat to self or others.^8^

Currently, little is known about the determinants of ALOS in LMICs.^5,7^ Similarly, there is no agreement on an acceptable recommended admission time for a psychiatric episode in LMICs.^5^ In this study, we investigated records of patients who were admitted to an acute psychiatric ward and investigated differences in demographic, clinical and system variables between patients who were admitted for longer than 21 days and patients who were discharged within 14 days. Sociodemographic factors included age, gender, level of education, nationality, employment and marital status, residence or accommodation and access to health insurance or medical aid.^3^ Clinical factors that may influence ALOS include diagnosis, past psychiatric history, duration and severity of the illness, reason for admission/current episode, legal status, medical/surgical/physical/psychiatric comorbidity and presence of extrapyramidal side effects.

### Aims and objectives

#### Aims

To evaluate measurable variables that are associated with a long duration of hospitalisation (>21 days) in an acute psychiatric unit at Biko Academic Hospital.

#### Objectives

The study investigated factors that influence ALOS in an acute psychiatric unit among psychotic mental healthcare users (MHCUs). The secondary objective was to identify common psychotic diagnosis related to the length of psychiatric hospitalisation.

## Research methods and design

### Study design

This was a comparative, quantitative case-control study.

### Setting

The research was conducted at Steve Biko Academic Hospital (SBAH) acute psychiatry ward situated in Pretoria, Gauteng, in South Africa.

### Study population and sampling strategy

Initially, the sample size consisted of 100 admitted patients with 28 files in the short stay group (SSG) and 72 files in the long stay group (LSG). However, only 82 patients’ files were included because some files were not available; diagnoses were not correctly recorded or the patients did not meet the inclusion criteria. Data were collected from the clinical files of MHCUs who met the inclusion criteria.

The inclusion criteria were MHCUs receiving inpatient care under legal status (voluntary, assisted and involuntary) at SBAH psychiatry unit above the age of 18 years with a clinical or psychiatric diagnosis presenting with any disorder with psychotic symptoms. Exclusion criteria were all psychiatric patients who were transferred to other medical or surgical disciplines. The MHCUs who spent less than 14 days in the ward were assigned the label SSG, and those who were admitted for more than 21 days in the ward were assigned the LSG title. We compared the demographic, clinical and health system variables between these two groups.

### Data collection

Data were recorded into a specially designed questionnaire. Data charting was completed by a medical professional who evaluated each clinical file, extracting patients’ age, psychiatric diagnosis, substance use history, choice of antipsychotic and whether the patient was discharged or transferred to another facility. This questionnaire was used to measure the psychiatric patient’s stay in hospital during admission, the factors associated with the length of hospitalisation and the reasons for transfer and referral to other facilities.

#### Data management and analysis

Data were collected and recorded on a data collection sheet. The assistance of a statistician was obtained in the form of a statistician’s report. Data were captured electronically onto Microsoft Excel by the researcher. Data were further analysed by a statistician using SAS version 9.4. Descriptive statistics, frequencies and percentages were calculated for categorical data, and medians and percentiles for numerical data. The Shapiro-Wilk test was performed to ensure normal distribution of numerical data.^9^ Analytical statistics called Fisher’s exact test was used to compare group proportions.^10^ A significance level (α) of 0.05 was used.

#### Ethical considerations

The study was approved by the Faculty of Health Sciences Research Ethics Committee of the University (ethics number [446/2020]). Permission was also obtained from the appropriate management executives to use the clinical files. Informed consent was obtained from patients; this was waived, as it was a retrospective file review.

## Results

**FIGURE 1:**
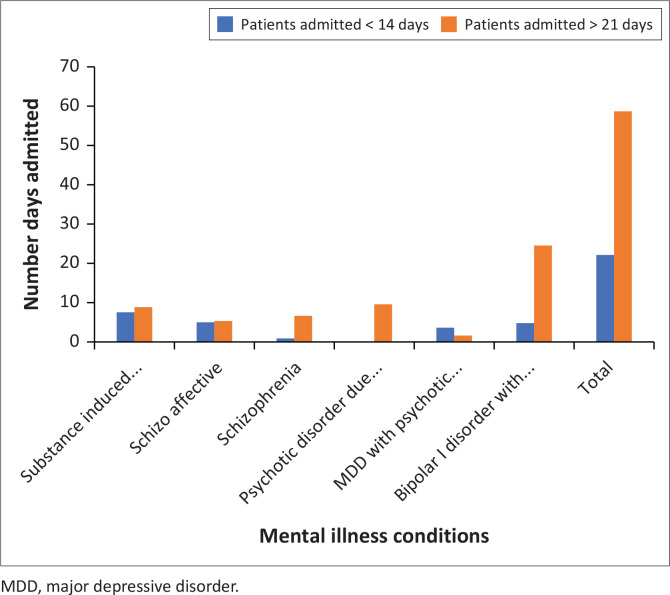
Patients admitted with psychosis symptoms.

The study selected 100 files and reviewed 82 patient files: 28.05% (*n* = 23) in SSG (<14 days) and 72% (*n* = 59) in LSG (> 21 days). The other files were invalid because of lack of information. More than 80% of patients were South African and nearly 30% of participants in the study had higher qualifications. There was no significant difference in terms of nationality or educational level among the two groups the results can be seen in Figure 1.

The age of participants was not normally distributed (SSG: *W* = 0.884, *p* = 0.0177; LSG *W* = 0.928, *p* = 0.0020). Consequently, median and interquartile range (IQR) of ages were reported. Participants in the SSG had a median age of 33 years (quartile IQR of 21 and quartile IQR 46), while participants in LSG had a median age of 35.5 years (lower IQR of 24 and upper IQR of 46). The oldest patient in LSG was 71 years, which was almost the same as the patient in SSG, who was 62 years. There was a significant difference in the age of patients between the SSG and LSG (*p* = 0.02).

In SSG, there were more men participants than women participants (men 78.3%, women 21.8%). While the LSG had equal proportions of men and women (men 49.2%, women 50.9%), there was a significant gender difference between SSG and LSG (*p* < 0.05).

In the SSG, 87% of patients were dominantly single compared to 64% of patients in LSG. The number of married people in the LSG was double that of single participants, and the number of widowed people in the LSG was tripled compared with the number of widowed people in SSG. Relationship status did not differ significantly between the two groups.

About 95% of patients from the SSG received support from their families, while 85% of patients in LSG received support from families (*p* < 0.05). Most of the patients had stable accommodation, and only about 8.5% in LSG were homeless. There were no homeless people in SSG. More than 60% of the patients were unemployed.

### Clinical variables

Legal status was similar for MHCUs in both groups. According to the Mental Health Care Act, patients can be admitted in one of the three categories, namely voluntary, assisted or involuntary.^11^ In both groups, only one patient was admitted voluntarily, and there were approximately equal proportions of assisted (SSG: 52% and LSG: 57%) and involuntary (SSG: 47% and LSG: 40%) admissions.

Short stay group had a higher proportion of patients who presented for the first time (index presentations = 39%), while 33% of patients in LSG were index presentations. Long stay group had a higher proportion of known MHCUs (66.10%) compared to SSG (56.62%). The two groups did not differ in terms of the number of the index cases or the number of known MHCUs.

The study identified 10 diagnoses in the patient files. The most common diagnosis in SSG was substance-induced psychotic disorder, and the most common diagnosis in LSG was bipolar I disorder with psychotic features (Table 1). The Fisher’s exact test showed that the SSG and the LSG had different proportions of psychotic disorders (*p* < 0.05).

**TABLE 1:** Most common diagnoses recorded among patients admitted to a psychiatric unit.

Diagnosis	Short stay group (%)	Long stay group (%)
Substance-induced psychotic disorder	34.8	18.6
Schizophrenia	21.7	13.6
Schizoaffective	4.4	11.9
Psychotic disorder because of another medical disorder	0	17
MDD with psychotic features	17.4	5.1
Bipolar I disorder with psychotic features	21.7	44.1

MDD, major depressive disorder.

Table 1 reflects the most common diagnoses recorded among patients admitted to a psychiatric unit. Short stay group refers to patients who were discharged within 14 days and LSG indicates patients who had long hospitalisations.

A large proportion of patients in SSG used illicit substances (73.9%) compared to LSG (44.1%) (*p* = 0.02). A larger proportion of patients in LSG had medical/surgical and psychiatric comorbidities (67.8%) compared to SSG (43.5%) (*p* < 0.04). The two groups had similar rates of second-generation antipsychotic use (SSG = 90%; LSG = 98.3%). More than 90% of patients (excluding patients who were index cases), irrespective of group, were known to have defaulted on their medication. Both groups had similar low incidence of complications from antipsychotic medications (SSG = 4.4%; LSG = 8.5%). More than 80% of patients were discharged from the acute unit in both groups.

### System or organisational variables

Only 16% of patients from both groups were severely ill and required longer-term care. These patients needed to be transferred to the next level of care, which is Weskoppies Psychiatric Hospital. On average, patients had to wait an additional 3 weeks before being transferred to the next level of care. No MHCUs were deported to other countries from the unit. There was no notable erratic drug supply that impacted LOS.

## Discussion

The study compared two groups of patients to establish differences between patients who had prolonged hospital stay beyond 21 days and patients who were discharged within 14 days. Patients who spent less time in the acute psychiatric unit tended to be young, medically healthy males presenting with acute substance-related disorders that were stabilised quickly.^12^

Long hospitalisation seemed to be more common among female patients who were presented with primary psychiatric disorders and different medical, surgical or obstetric comorbidities.

Family support was associated with shorter hospitalisation.^13^ A large proportion of patients were single but displayed sufficient family support, which was associated with early hospitalisation and continuation of care beyond psychiatric hospitalisation discharge.^14^

Depending on the severity of illness, having family support results in a quicker turnaround time because of family support and care beyond psychiatric hospitalisation.^14^

Although homelessness did not emerge as the main challenge in this study, it was noted among the patients in the long hospitalisation group that a large proportion of patients were also admitted under assisted or involuntary sections. The discharging of homeless patients is a challenge because it requires that social workers organise a suitable place that is licensed to look after MHCUs. Delaying the discharge of these patients results in ‘bed blocking’, where stable patients occupy beds in acute facilities when they should be transferred to a community mental healthcare facility. In this study, the problem was inadequate or unavailable community-based healthcare facilities.

Long hospitalisation seemed to be associated with certain conditions. In this study, substance-induced psychosis was more common in the SSG than in the long hospitalisation group. Surprisingly, patients with schizophrenia were more commonly diagnosed in the SSG than in the long hospitalisation group, which could be attributed to patients being transferred or discharged earlier than 2 weeks because medication could have been introduced at the referral site prior to admission. As the study group had older patients with more comorbidities, it made sense that psychosis because of another medical condition was common in the long hospitalisation patients.^15^ The most common medical conditions among psychiatric patients included side effects because of second-generation antipsychotics such as hypertension, dyslipidaemia and diabetes mellitus type 2. Other medical conditions such as human immunodeficiency virus (HIV) infection may have caused psychotic symptoms. This study took place at an academic institution where medical diagnoses are accompanied by intensive consultation with the relevant discipline to ensure the patient is initiated on the latest treatments. This process tends to add more days of in-hospital care or follow-up investigations or the need to wait for specialist feedback.

In the case where patients required rapid tranquillisation, doctors in this setting primarily used first-generation antipsychotic medication as Haloperidol 5 mg intramuscularly in combination with a short-acting benzodiazepine such as Lorazepam 4 mg.^16^ Intramuscular first-generation antipsychotic depots were introduced for poorly compliant patients and at the patient’s request because they found oral titrations tedious and difficult to take consistently. The long-term treatment on discharge was usually second-generation oral medications (Risperidone or Olanzapine). Although there was a period in 2019 where Olanzapine was out of stock from the depot supplier, few patients were affected. Erratic drug supply and the lack of community mental programmes that address social problems remain a struggle at varying severities globally.^5^

### Limitations

There was no validated scale or tool. Some clinical files were excluded because of errors in the recorded final diagnoses or missing data, thus reducing the sample size. Clinicians could not accurately record disease severity, which could explain the reasons for longer hospitalisation or the need for transfer to higher levels of care. Poor record-keeping also reduced the amount of raw data that were available for analysis and interpretation.

### Strengths

To our knowledge, this is the first study to examine the factors affecting long hospitalisation in MHCU at an acute psychiatry unit in South Africa.

## Conclusion

Shorter stays in psychiatry wards at SBAH were associated with younger males presenting with conditions that require rapid stabilisation. Long stay was found to be associated with older females with primary psychotic disorders and comorbidities. In this setting, a digital system that records data to avoid missing files and loss of information is needed. Although deinstitutionalisation remains a challenge in South Africa, patients’ family members assist with continuation of care; hence, 95% of patients in the SSG had family support.
